# ^1^H, ^13^C and ^15^N resonance assignments for the microtubule-binding domain of the kinetoplastid kinetochore protein KKT4 from *Trypanosoma brucei*

**DOI:** 10.1007/s12104-020-09968-1

**Published:** 2020-07-21

**Authors:** Patryk Ludzia, Bungo Akiyoshi, Christina Redfield

**Affiliations:** Department of Biochemistry, University of Oxford, South Parks Road, Oxford, OX1 3QU UK

**Keywords:** KKT4, Kinetoplastid, Kinetochore, Trypanosomes, NMR resonance assignments

## Abstract

KKT4 is a kinetoplastid-specific microtubule-binding kinetochore protein that lacks significant similarity to any known kinetochore or microtubule-binding proteins. Here we present the ^1^H, ^13^C and ^15^N resonance assignments for several fragments from the microtubule-binding domain of KKT4 (KKT4^115–343^) from *Trypanosoma brucei*. These assignments provide the starting point for detailed investigations of the structure, dynamics and interactions of the microtubule-binding region of KKT4.

**Electronic supplementary material:**

The online version of this article (10.1007/s12104-020-09968-1) contains supplementary material, which is available to authorized users.

## Biological context

During chromosome segregation, cells must accurately transmit their genetic material into two daughter cells in an organised manner. Errors in this process lead to genetic abnormalities that can lead to cancer or cell death. Chromosome segregation is therefore a critical requirement for the survival and development of all organisms (McIntosh 2016). In eukaryotes, a key structure involved in this process is the kinetochore, a dynamic protein complex that assembles onto centromeric DNA of each chromosome and captures spindle microtubules (Brinkley and Stubblefield 1966; Cheeseman 2014). Many kinetochore components, such as CENP-A and Ndc80, are widely conserved among eukaryotes (Biggins 2013; Cheeseman and Desai 2008; Meraldi et al. 2006; Santaguida and Musacchio 2009; van Hooff et al. 2017) and it was thought that all eukaryotes utilise the same set of kinetochore proteins.

However, none of the canonical kinetochore proteins has been found in kinetoplastids, an evolutionarily divergent group of unicellular flagellated eukaryotes including parasitic trypanosomatids (e.g. *Trypanosoma brucei*,* Trypanosoma cruzi*, and *Leishmania* species). Instead, a number of unique kinetochore proteins, KKT1–25 and KKIP1–12, were identified in *T. brucei* (Akiyoshi 2016; Akiyoshi and Gull 2014; Brusini et al. 2019; D’Archivio and Wickstead 2017; Nerusheva and Akiyoshi 2016; Nerusheva et al. 2019). No significant similarity to conventional kinetochore proteins is found in these proteins and very little is known about their structure, function and interactions.

We recently showed that the KKT4 protein, and in particular a fragment containing residues 115–343, has microtubule-binding activities in vitro (Llauro et al. 2018). Further investigation demonstrated that KKT4 can track depolymerising microtubule tips and form load-bearing attachments with microtubules. Therefore, KKT4 has all the functions required for a microtubule-binding kinetochore protein. Interestingly, sequence analysis of the protein failed to reveal significant similarity to any known kinetochore or microtubule-binding proteins. Structural characterisation of KKT4 is therefore essential for understanding the molecular mechanism of its function. Here we present ^1^H, ^13^C and ^15^N resonance assignments for the microtubule-binding region of the kinetoplastid kinetochore protein KKT4 (KKT4^115–343^) from *T. brucei*. These NMR assignments provide the starting point for detailed investigations of the structure and dynamics of KKT4.

## Methods and experiments

### Protein expression and purification

KKT4 fragments characterised in this study were amplified from *T. brucei* genomic DNA that was cloned into the pNIC28-Bsa4 expression vector using a ligation-independent cloning method (Gileadi et al. 2008). Transformed *E. coli* BL21(DE3) cells were plated on agar plates containing 50 µg/ml kanamycin and incubated at 37 °C overnight. After overnight incubation, a few colonies were inoculated into 5 ml of 2xTY medium containing 50 µg/ml kanamycin and grown at 37 °C for 6 h. Next, 50 ml of M9 minimal medium containing 50 µg/ml kanamycin supplemented with 1 g/l ^15^NH_4_Cl and 4 g/l [^13^C]-*D*-glucose (CIL) as the sole nitrogen and carbon sources was inoculated with 500 μl of bacterial culture. Cell growth was continued overnight at 37 °C. Next, 5 ml of overnight culture was inoculated into 1 l of M9 minimal medium supplemented with 1 g/l ^15^NH_4_Cl, 4 g/l [^13^C]-*D*-glucose and 50 µg/ml kanamycin. Cells were grown at 37 °C to an OD_600_ of ∼ 0.8. Protein expression was induced by 0.4 mM IPTG and incubated overnight at 16 °C with shaking (200 rpm). Expression of uniformly labelled ^2^H/^13^C/^15^N-KKT4^145–232^ was facilitated by cell growth in D_2_O-based M9 minimal medium, with 1 g/l ^15^NH_4_Cl, 4 g/l [^13^C]-*D*-glucose and 50 µg/ml kanamycin, according to the protocol described in (Tugarinov et al. 2006).

Cells were pelleted at 3400*g* at 4 °C and resuspended in lysis buffer (50 mM sodium phosphate pH 7.5, 500 mM NaCl, and 10% glycerol) supplemented with protease inhibitors (20 μg/ml leupeptin, 20 μg/ml pepstatin, 20 μg/ml E-64, 2 mM benzamidine, and 0.4 mM PMSF) and 0.5 mM TCEP. All subsequent extraction steps were performed at 4 °C. Cell lysis was facilitated by mechanical cell disruption (French press, 1 passage at 20,000 psi). Lysed cells were spun at 48,000*g* for 30 min and the supernatant was loaded on a gravity column with TALON beads (Takara Clontech) pre-equilibrated in lysis buffer. After loading, the beads were washed extensively with lysis buffer and proteins were eluted with elution buffer (50 mM sodium phosphate pH 7.5, 500 mM NaCl, 10% glycerol, 250 mM imidazole, 0.5 mM TCEP). To remove the N-terminal 6xHis tag, the proteins were incubated with TEV protease and dialysed overnight against buffer containing 25 mM sodium phosphate pH 7.5, 250 mM NaCl, 5% glycerol, 5 mM imidazole and 0.5 mM TCEP. After overnight incubation, samples were diluted with 25 mM HEPES pH 7.5 with 0.5 mM TCEP (buffer A) to the final NaCl concentration of 50 mM and loaded on a cation exchange RESOURCE S column (GE Healthcare), pre-equilibrated with 5% of buffer B (25 mM HEPES pH 7.5, 1 M NaCl and 0.5 mM TCEP). Proteins were eluted from the column with a linear gradient from 5 to 100% of buffer B. Fractions containing KKT4 fragments were pooled, concentrated and loaded on a gel filtration column SD200 or SD75 16/60 (GE Healthcare) to purify further and buffer exchanged into 25 mM HEPES pH 7.5, 150 mM NaCl and 0.5 mM TCEP. The samples were concentrated using a 10-kD MW Amicon concentrator (Millipore), and aliquots were flash-frozen in liquid nitrogen and stored at − 80 °C.

### NMR spectroscopy

^15^N or ^13^C/^15^N-labelled samples of KKT4^115–174^, KKT4^145–232^ and KKT4^115–343^ were used for resonance assignment using standard triple-resonance protocols (Redfield 2015). For KKT4^145–232^ a ^2^H/^13^C/^15^N-labelled sample was also required. All samples contained 300–500 µM protein in a 25 mM HEPES buffer at pH 7.2 with 150 mM NaCl, 0.5 mM TCEP and 5% D_2_O. NMR experiments were carried out at 20 °C for KKT4^115–174^ and KKT4^115–343^ and at 30 °C for KKT4^145–232^, using 750 and 950 MHz spectrometers equipped with Oxford Instruments Company magnets, Bruker Avance III HD consoles and 5 mm TCI CryoProbes.

The KKT4^115–174^, KKT4^145–232^ and KKT4^115–343^ samples were initially characterised using 2D ^1^H–^15^N HSQC (Kay et al. 1992; Palmer et al. 1991; Schleucher et al. 1994) SOFAST-HMQC (Schanda and Brutscher 2005) and BEST-TROSY (Lescop et al. 2007; Schulte-Herbruggen and Sorensen 2000) experiments. The latter two experiments allow rapid data collection or more extensive signal averaging than the HSQC. In crowded regions of the spectra, particularly for KKT4^115–343^, the resolution in the SOFAST-HMQC was significantly worse than that in the BEST-TROSY. Many residues in all the KKT4 constructs, particularly in KKT4^145–232^, gave rise to relatively weak peaks due to unfavourable relaxation properties (Supplementary Fig. 1); for these residues the highest quality data were obtained using the BEST-TROSY experiment. In order to be consistent between the three constructs, the assignments for all three are illustrated below using BEST-TROSY spectra.

Resonance assignments for KKT4^115–174^, KKT4^145–232^ and KKT4^115–343^ were obtained using 2D ^1^H–^13^C HSQC and ^1^H–^15^N BEST-TROSY experiments and 3D experiments including ^15^N-edited NOESY-HSQC, ^15^N-edited TOCSY-HSQC, HNCA, CBCANH, CBCA(CO)NH, HNCACB, HN(CO)CACB, HNCO, HN(CA)CO, HBHA(CBCACO)NH, HCA(CO)N, HCAN, (H)N(CA)NNH, (H)N(COCA)NNH, (H)CC(CO)NH, and HCCH-TOCSY. For KKT4^115–174^ and KKT4^145–232^ BEST-TROSY versions of the HNCA, HNCO, HN(CA)CO, HNCACB and HN(CO)CACB experiments were collected (Lescop et al. 2007; Schulte-Herbruggen and Sorensen 2000); for KKT4^145–232^ these incorporated ^2^H-decoupling. All 3D experiments were collected with 25% non-uniform sampling in the two indirect dimensions using standard Bruker sampling schedules. 2D NMR data were processed using NMRPipe (Delaglio et al. 1995) and 3D NUS data were processed with the hmsIST software (Hyberts et al. 2012) and NMRPipe. Spectra were analysed and assignments recorded using CcpNmr Analysis version 2.4 (Vranken et al. 2005). ^1^H and ^13^C chemical shifts were referenced using DSS and ^15^N chemical shifts were referenced indirectly. Details of the specific experiments and sample conditions used for each construct can be found in the BMRB deposition files.

### Extent of assignments and data deposition

**Fig. 1:**
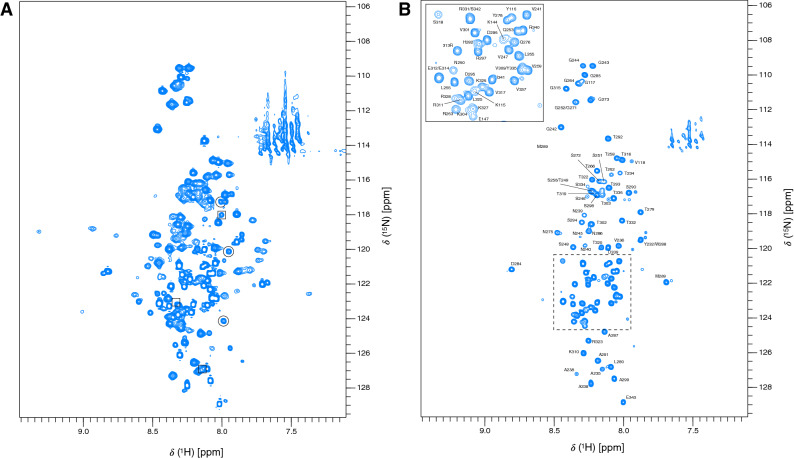
750 MHz ^1^H–^15^N BEST-TROSY spectra of KKT4^115–343^ in 25 mM HEPES, 150 mM NaCl and 0.5 mM TCEP (95% H_2_O/5% D_2_O), at pH 7.2, 20 °C. **a** This spectrum is contoured at a low level to highlight the range of peak intensities observed for KKT4^115–343^. **b** The peak assignments for backbone amides of the C-terminal region (residues 231–343) of KKT4^115–343^ are annotated. The inset is an expansion of the area outlined with dashed lines. Peaks in the region of 111–114 ppm and upfield of ~ 7.6 ppm are artefacts in the BEST-TROSY arising from incomplete cancellation of signals from the side chain amides of Asn and Gln which have not been assigned. The three circled peaks in **a** correspond to Y278 (^15^N = 120 ppm), T279 (^15^N = 117 ppm) and L280 (^15^N = 124 ppm) in a minor species involving P281 in a *cis* peptide bond. The three peaks in squares in **a** correspond to Y278 (^15^N = 123 ppm), T279 (^15^N = 118 ppm) and L280 (^15^N = 127 ppm) in a minor species involving P277 in a *cis* peptide bond

The ^1^H–^15^N BEST-TROSY spectrum of KKT4^115–343^ shows a large variation in peak intensities (Fig. 1a). Approximately 160 peaks are observed; this is less than expected for the 218 non-proline residues. For ~ 30 of the weakest peaks no ^13^C correlations were observed in triple resonance experiments making assignment of these impossible. The range of peak intensities for KKT4^115–343^ suggests a mixture of structured and disordered regions and/or differences in backbone dynamics on a µs–ms timescale leading to significant broadening and disappearance of many of the expected peaks. The strongest peaks visible in the ^1^H–^15^N BEST-TROSY spectrum of KKT4^115–343^ were assigned at pH 7.2 and 20 °C (Fig. 1b). ^1^H^N^ and ^15^N backbone resonances for 90 of the 218 non-proline residues of KKT4^115–343^ were assigned. These all correspond to residues between N231 and E343, the C-terminal residue. In this region of the sequence, 87.4% of ^1^H^N^ and 88.5% of ^15^N were assigned; further statistics for the extent of ^1^H and ^13^C assignments are presented in Table 1. Most of the missing ^1^H^N^/^15^N assignments correspond to Asn/Ser/Thr residues which either have fast intrinsic solvent exchange rates or are located in regions of low sequence complexity (for example, TTTSS). In addition to backbone resonance assignments, some side chain ^1^H and ^13^C assignments were obtained from HBHA(CBCACO)NH, (H)CC(CO)NH and ^15^N-TOCSY-HSQC experiments (Table 1).

**Table 1:** Extent of assignment for KKT4^115−343^, KKT4^115−174^ and KKT4^145−232^

	Percent assigned					
Sample^a^	^1^H^N^/^15^N^b^	^13^Cʹ	^1^Hα/^13^Cα	^1^Hβ/^13^Cβ	^1^Hγ/^13^Cγ^c^	^1^Hδ/^13^Cδ^d^
KKT4^115−343^	87.4/88.5	87.6	91.8/91.2	87.4/94.2	−/64.3	−/50.0
KKT4^115−174^	100/98.3	90.0	100/100	68.0/87.5	38.3/50.0	43.8/45.5
KKT4^145−232^	96.6/95.5	96.6	−/98.9	−/89.5	−/−	−/−

^a^All three proteins contain an N-terminal Ser-Met sequence, which remains after TEV protease cleavage, preceding the native sequence; assignments for these residues were not carried out. Assignment statistics are for residues of the native sequence. For KKT4^115−343^, assignment statistics are reported for residues 231–343 only

^b^Backbone ^15^N statistics include proline nitrogens

^c^Gamma carbons from Asp, Asn, His, Phe, Tyr and Trp, which do not have attached ^1^H and are generally not assigned, are not included in the statistics

^d^Delta carbons from Glu, Gln and Trp (δ2), which do not have attached ^1^H and are generally not assigned, are not included in the statistics

In order to obtain assignments for residues in the N-terminal part of KKT4, NMR data were collected for a shorter fragment, KKT4^115–232^, containing the residues that could not be assigned in KKT4^115–343^. However, the spectrum of KKT4^115–232^ did not show the expected number of peaks (not shown). To overcome this, two shorter overlapping constructs, KKT4^115–174^ and KKT4^145–232^, were used. KKT4^115–174^ has been shown previously to bind to microtubules, albeit less efficiently than KKT4^115–343^ (Llauro et al. 2018). KKT4^145–232^ has been identified as a stable fragment in trypsin digests of KKT4^115–343^ (Supplementary Fig. 2). The ^1^H–^15^N BEST-TROSY spectra of KKT4^115–174^ and KKT4^145–232^ show the expected number of peaks, more uniform peak intensities and better dispersion of ^1^H^N^ chemical shifts than observed for KKT4^115–343^.

**Fig. 2:**
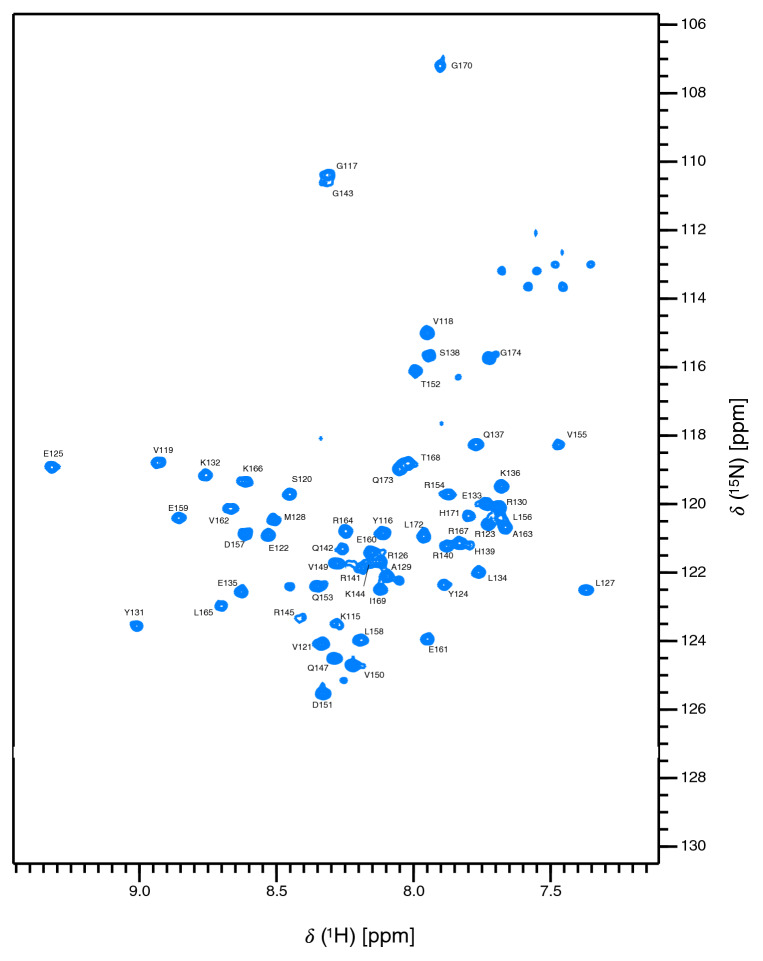
750 MHz ^1^H–^15^N BEST-TROSY spectrum of KKT4^115–174^ in 25 mM HEPES, 150 mM NaCl and 0.5 mM TCEP (95% H_2_O/5% D_2_O), at pH 7.2, 20 °C. The peak assignments for backbone amides are annotated. Peaks in the region of 111–114 ppm and upfield of ~ 7.6 ppm are artefacts in the BEST-TROSY arising from incomplete cancellation of signals from the side chain amides of Gln; these NH_2_ groups have been assigned in the HSQC spectrum shown in Supplementary Fig. 3A

**Fig. 3:**
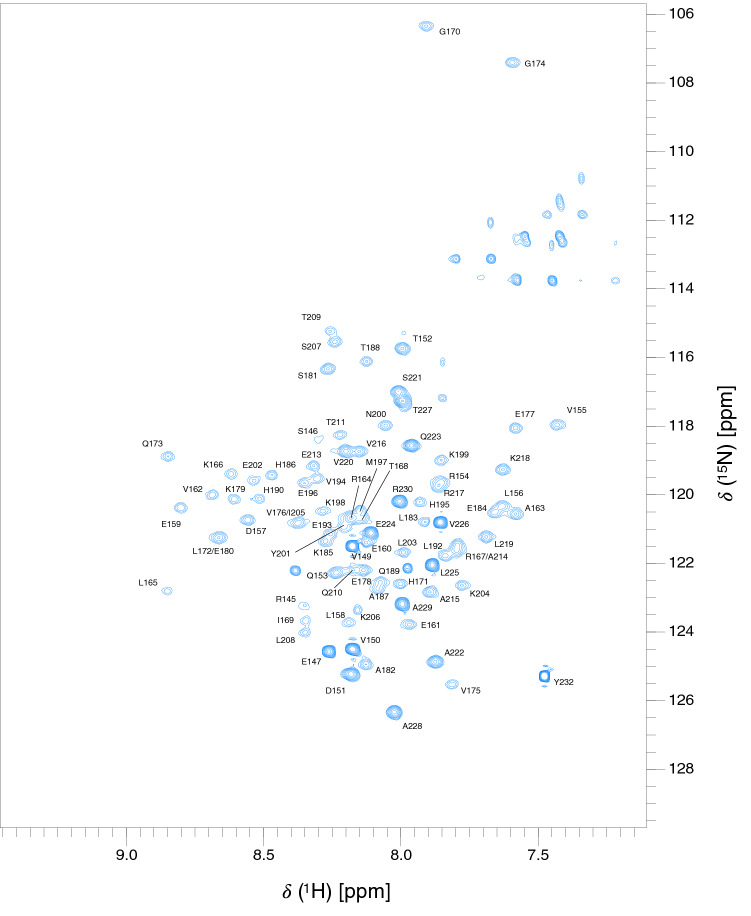
**7**50 MHz ^1^H–^15^N BEST-TROSY spectrum of KKT4^145–232^ in 25 mM HEPES, 150 mM NaCl and 0.5 mM TCEP (95% H_2_O/5% D_2_O), at pH 7.2, 30 °C. The peak assignments for backbone amides are annotated. Peaks in the region of 111–114 ppm and upfield of ~ 7.6 ppm are artefacts in the BEST-TROSY arising from incomplete cancellation of signals from the side chain amides of Asn and Gln; these NH_2_ groups have been assigned in the HSQC spectrum shown in Supplementary Fig. 3B

For KKT4^115–174^ extensive backbone and side-chain assignments were completed at pH 7.2 and 20 °C (Fig. 2). This includes ^1^H/^13^C assignments for 82.6% of methyl groups and all three tyrosine side chains; detailed assignment statistics are presented in Table 1. Assignment of the KKT4^145–232^ spectrum proved more challenging; although the expected number of peaks were observed in the BEST-TROSY spectrum, triple resonance data were of poor quality. Deuteration of KKT4^145–232^ was required in addition to ^13^C/^15^N labelling and triple resonance spectra were collected at 30 °C (Fig. 3). Because of deuteration, only ^1^H^N^, ^15^N, ^13^Cα, ^13^Cβ and ^13^Cʹ assignments were possible for KKT4^145–232^; assignment statistics are presented in Table 1. For both KKT4^115–174^ and KKT4^145–232^, assignments for all Asn and Gln side chain NH_2_ groups were obtained using HSQC and triple-resonance spectra (Supplementary Fig. 3). The quality of the spectra obtained for KKT4^115–174^ and KKT4^145–232^ are significantly poorer than would be expected for monomeric, globular proteins of 60 and 88 residues. This suggests that these constructs may exist as oligomers and/or have overall shapes that give rise to anisotropic tumbling resulting in challenging relaxation properties; this will be explored in future studies of their structure and dynamics.

Assignments were obtained for all ten proline residues in the C-terminal region of KKT4^115–343^. This included ^15^N assignments obtained from HCA(CO)N and HCAN experiments which correlate the proline ^15^N with the preceding ^1^Hα/^13^Cα, and the proline ^15^N with both the preceding ^1^Hα/^13^Cα and the proline ^1^Hα/^13^Cα and ^1^Hδ/^13^Cδ, respectively. A previous study of the ID4 linker region of CREB-binding protein showed a clustering of peaks observed in the proline-fingerprint region of 2D CON spectra according to the type of residue preceding the proline (highlighted for GP, LP, PP, SP, TP and VP motifs) (Murrali et al. 2018)*.* For KKT4^115–343^, the ^13^Cʹ(i-1)/^15^N(i) chemical shifts for G273/P274, L280/P281, T320/P321 and T332/P333 fall within the regions highlighted for these motifs in ID4 (Supplementary Table 1). Murrali et al*.* observe two clusters each for the AP and QP motifs; in both cases the proline ^15^N chemical shift is ~ 1.5–2 ppm further downfield if the proline is followed by a second proline (APP/QPP instead of APX/QPX) (Murrali et al. 2018). For KKT4^115–343^, the chemical shifts for A299/P300, Q253/P254 and Q276/P277, which are not followed by proline, fall within the regions observed for the APX and QPX motifs in ID4 (Supplementary Table 1).

Analysis of the proline ^13^Cβ and ^13^Cγ chemical shifts shows that the major conformation for all ten prolines involves a *trans* peptide bond (Schubert et al. 2002). However, a number of weak peaks are observed in the BEST-TROSY spectrum and in many cases these can be assigned to residues in the vicinity of a *cis* proline. For example, the three peaks highlighted with circles in Fig. 1a have been assigned to the sequence Y278-T279-L280 where P277 and P281 are in the *trans* and *cis* conformations, respectively; while the three peaks highlighted with squares are assigned to Y278-T279-L280 where P277 and P281 are in the *cis* and *trans* conformations, respectively. Analysis of the peak intensities indicates populations of ~ 10% and ~ 13% for the P277_*cis*_ and P281_*cis*_ conformers, respectively. A recent study of proline conformation in the intrinsically disordered protein Osteopontin suggested that *cis* proline populations of over 10% were only observed for proline residues directly preceded or followed by an aromatic residue (Phe or Trp) (Mateos et al. 2020)*.* Interestingly, in KKT4^115–343^, P277 and P281 are also followed by aromatic residues, Y278 and H282.

The ^1^H, ^13^C and ^15^N chemical shift assignments presented here for KKT4^115–174^, KKT4^145–232^ and KKT4^115–343^ have been deposited in the BioMagResBank (http://www.bmrb.wisc.edu) under the accession numbers 50215, 50228 and 50229, respectively. These assignments provide the starting point for detailed investigations of the structure, dynamics and interactions of the microtubule-binding domain of KKT4 (KKT4^115–343^) from *T. brucei*.

## Electronic supplementary material

Below is the link to the electronic supplementary material.

Supplementary file1 (PDF 359 kb)

Supplementary file2 (PDF 156 kb)

Supplementary file3 (PDF 321 kb)

Supplementary file4 (PDF 49 kb)
