# Supplementary material for: 1H, 13C and 15N resonance assignments for the microtubule-binding domain of the kinetoplastid kinetochore protein KKT4 from Trypanosoma brucei
Source: Biomol NMR Assign. 2020 Jul 21;14(2):309–15. doi: 10.1007/s12104-020-09968-1 (PMC7462909; doi:10.1007/s12104-020-09968-1)
Supplement: Supplementary file 1 — Supplementary file1 (PDF 359 kb) [file 12104_2020_9968_MOESM1_ESM.pdf]

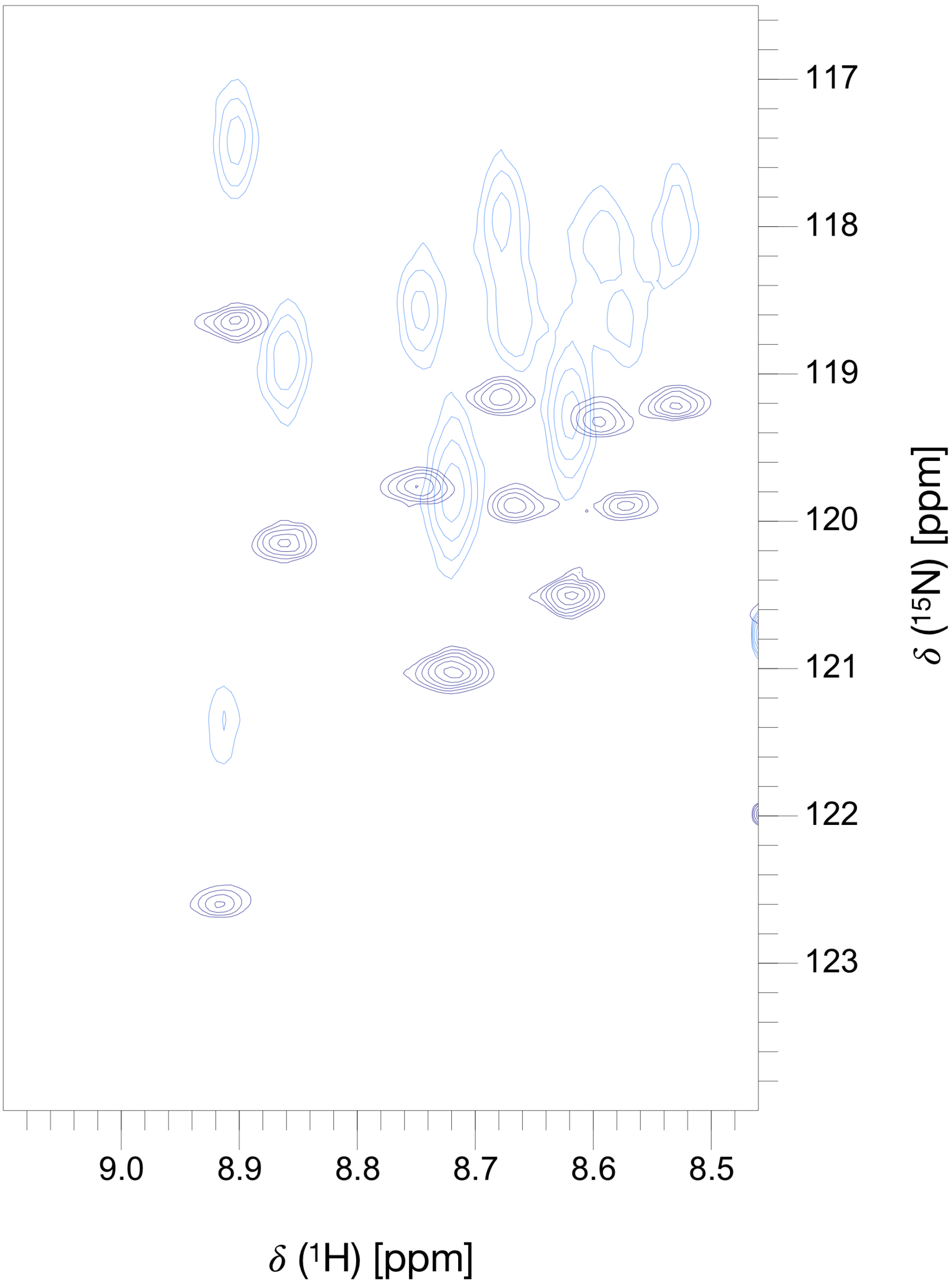

Supplementary Figure 1: Comparison of 750 MHz ^{1}H-^{15}N BEST-TROSY and semi-BEST-TROSY spectra of KKT4$^{145–232}$. A small region of the BEST-TROSY (dark blue) and semi-BEST-TROSY (light blue) spectra of KKT4$^{145-232}$, in 25 mM HEPES, 150mM NaCl and 0.5 mM TCEP (95% H_2O/5% D_2O), at pH 7.2, 30 °C, are overlaid. Peaks in the two spectra are offset in the ^{15}N dimension by the one-bond $^{1}H^{N}$-^{15}N coupling constant. Peaks in the BEST-TROSY spectrum show much narrower ^{15}N linewidths than observed in the semi-BEST-TROSY spectrum indicating that under the conditions being studied, KKT4$^{145-232}$ shows a significant 'TROSY' effect due to a long effective rotational correlation time.
