# Supplementary material for: 1H, 13C and 15N resonance assignments for the microtubule-binding domain of the kinetoplastid kinetochore protein KKT4 from Trypanosoma brucei
Source: Biomol NMR Assign. 2020 Jul 21;14(2):309–15. doi: 10.1007/s12104-020-09968-1 (PMC7462909; doi:10.1007/s12104-020-09968-1)
Supplement: Supplementary file 2 — Supplementary file2 (PDF 156 kb) [file 12104_2020_9968_MOESM2_ESM.pdf]

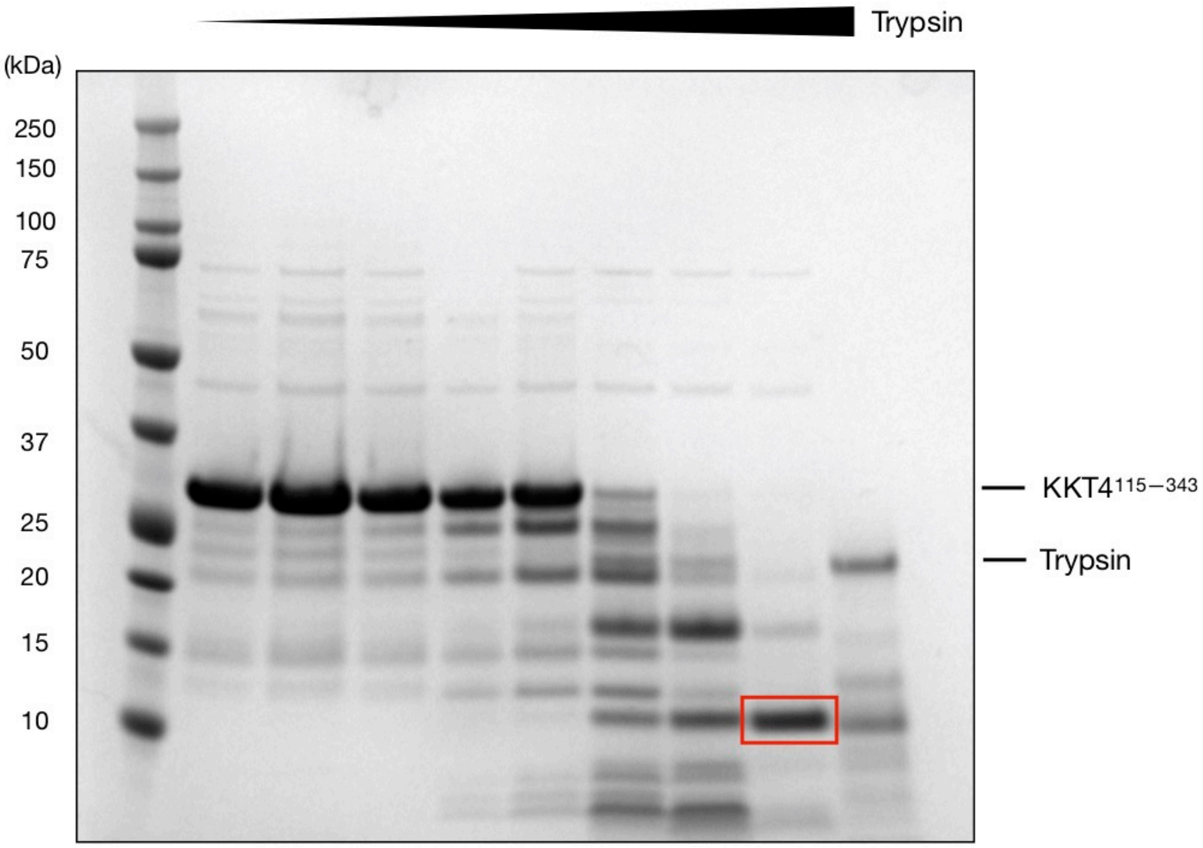

Supplementary Figure 2: The *in vitro* limited proteolysis of KKT4$^{115–343}$

Samples containing 20 µg of KKT4$^{115–343}$ in 50 mM sodium phosphate pH 7.5, 200 mM NaCl and 0.5 mM TCEP were mixed with increasing amounts (0, 0.002, 0.004, 0.02, 0.04, 0.2, 0.4, 2 and 200 µg – lanes 2-10) of trypsin in 25 mM Tris pH 8.0, 2 mM $CaCl_2$ in a 1:1 v/v ratio. Following incubation on ice for 1 hour, samples were analysed using SDS-PAGE to assess the degree of proteolysis. The band highlighted in red, identified following incubation with 2 µg trypsin, was cut out and subjected to mass spectrometry analysis. This revealed that the trypsin-resistant fragment corresponds to residues 145–230 of KKT4 (KKT4$^{145–230}$).
