# Supplementary material for: 1H, 13C and 15N resonance assignments for the microtubule-binding domain of the kinetoplastid kinetochore protein KKT4 from Trypanosoma brucei
Source: Biomol NMR Assign. 2020 Jul 21;14(2):309–15. doi: 10.1007/s12104-020-09968-1 (PMC7462909; doi:10.1007/s12104-020-09968-1)
Supplement: Supplementary file 3 — Supplementary file3 (PDF 321 kb) [file 12104_2020_9968_MOESM3_ESM.pdf]

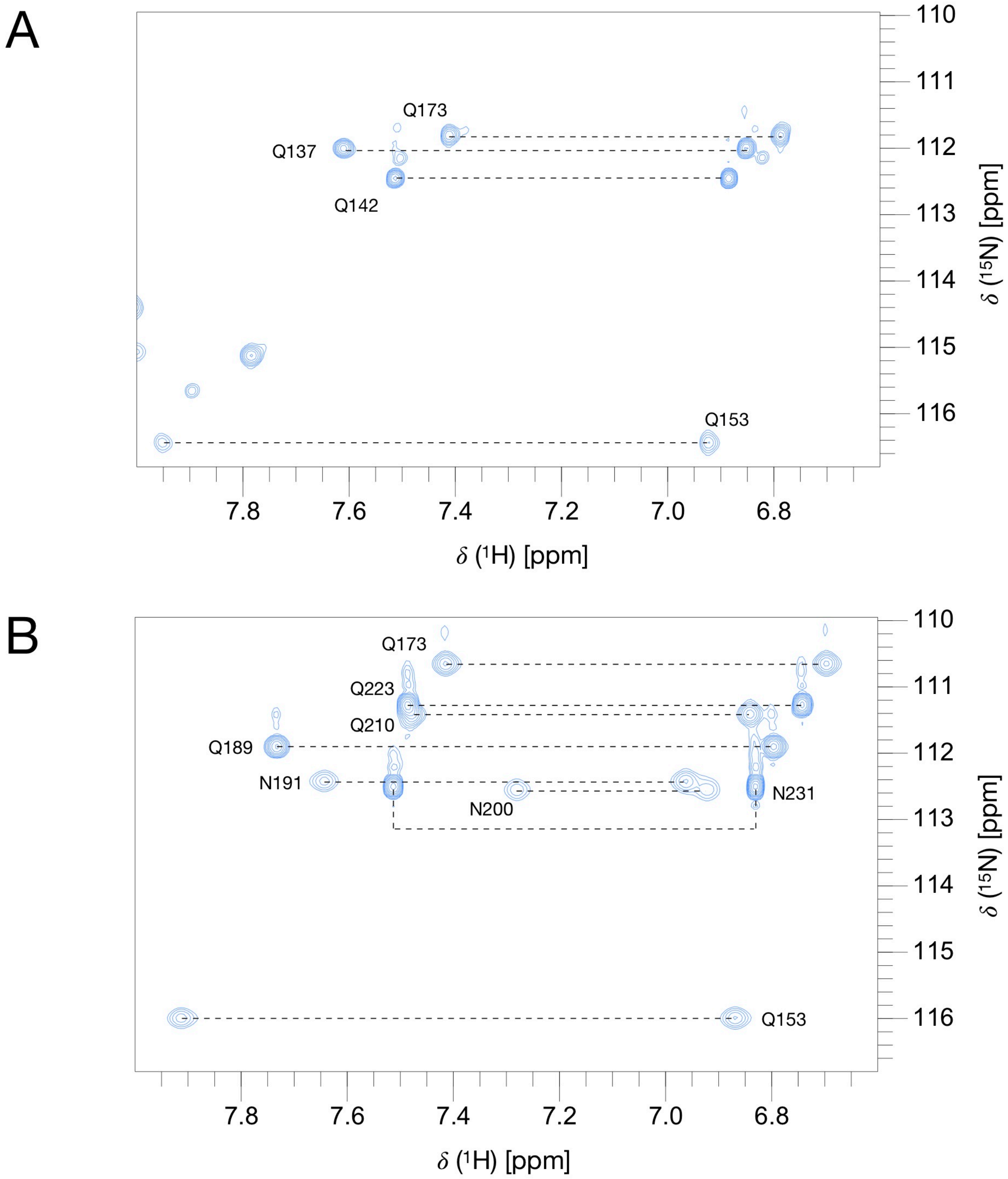

Supplementary Figure 3: Expansion of ^{1}H-^{15}N HSQC spectra showing side chain asparagine and glutamine NH_2 assignments. (A) HSQC spectrum of KKT4$^{115–174}$ in 25 mM HEPES, 150mM NaCl and 0.5 mM TCEP (95% H_2O/5% D_2O), at pH 7.2, 20 °C. Peak assignments for the four Gln side chain NH_2 are annotated. (B) HSQC spectrum of KKT4$^{145–232}$ in 25 mM HEPES, 150mM NaCl and 0.5 mM TCEP (95% H_2O/5% D_2O), at pH 7.2, 30 °C. Peak assignments for the two Asn and five Gln side chain NH_2 are annotated.
