# Supplementary material for: 1H, 13C and 15N resonance assignments for the microtubule-binding domain of the kinetoplastid kinetochore protein KKT4 from Trypanosoma brucei
Source: Biomol NMR Assign. 2020 Jul 21;14(2):309–15. doi: 10.1007/s12104-020-09968-1 (PMC7462909; doi:10.1007/s12104-020-09968-1)
Supplement: Supplementary file 4 — Supplementary file4 (PDF 49 kb) [file 12104_2020_9968_MOESM4_ESM.pdf]

Supplementary Table 1

^{13}C′(i-1) and ^{15}N(i) chemical shifts for Xxx-Pro motifs in KKT4$^{115\text{-}343}$

Residue Pair	^{13}C′ (i-1) (ppm)	^{15}N Pro(i) (ppm)
Q253-P254	174.0	137.1
G273-P274	172.0	133.9
Q276-P277	173.9	136.8
L280-P281	175.1	135.7
H282-P283	174.0	138.2
A299-P300	175.3	135.8
T320-P321	172.8	139.3
R323-P324	173.9	136.8
N329-P330	-	136.6
T332-P333	172.8	139.0
